# The Natural-Mineral-Based Novel Nanomaterial IFMC Increases Intravascular Nitric Oxide without Its Intake: Implications for COVID-19 and beyond

**DOI:** 10.3390/nano10091699

**Published:** 2020-08-29

**Authors:** Tomohiro Akiyama, Takamichi Hirata, Takahiro Fujimoto, Shinnosuke Hatakeyama, Ryuhei Yamazaki, Tomohiro Nomura

**Affiliations:** 1 Advanced Research Laboratories, Tokyo City University, Tokyo 158-8557, Japan; fujimoto@clinic-f.com; 2 Graduate School of Information Technology, Kobe Institute of Computing, Kobe 650-0001, Japan; 3 Graduate School of Education, Kyoto University, Kyoto 606-8501, Japan; 4 Graduate School of Global Environmental Studies, Sophia University, Tokyo 102-8554, Japan; 5 Graduate School of Integrative Science and Engineering, Electrical Engineering and Chemistry, Tokyo City University, Tokyo 158-8557, Japan; g1981258@tcu.ac.jp (S.H.); g1981268@tcu.ac.jp (R.Y.); 6 Clinic F Laser Medicine & Surgery, Tokyo 102-0083, Japan; 7 Osaka City University, Osaka 558-8585, Japan; t.nomura@abeam.ocn.ne.jp

**Keywords:** SARS-CoV-2, COVID-19, natural-mineral-based nanomaterial, nitric oxide, haemoglobin, blood flow promotion

## Abstract

There are currently no promising therapy strategies for either the treatment or prevention of novel coronavirus disease 2019 (COVID-19), despite the urgent need. In addition to respiratory diseases, vascular complications are rapidly emerging as a key threat of COVID-19. Existing nitric oxide (NO) therapies have been shown to improve the vascular system; however, they have different limitations in terms of safety, usability and availability. In light of this, we hypothesise that a natural-mineral-based novel nanomaterial, which was developed based on NO therapy, might be a viable strategy for the treatment and prevention of COVID-19. The present study examined if it could induce an increase of intravascular NO, vasodilation and the consequent increase of blood flow rate and temperature in a living body. The intravascular NO concentration in the hepatic portal of rats was increased by 0.17 nM over 35.2 s on average after its application. An ultrasonic Doppler flow meter showed significant increases in the blood flow rate and vessel diameter, but no difference in the blood flow velocity. These were corroborated by measurements of human hand surface temperature. To our knowledge, this result is the first evidence where an increase of intravascular NO and vasodilation were induced by bringing a natural-mineral-based nanomaterial into contact with or close to a living body. The precise mechanisms remain a matter for further investigation; however, we may assume that endothelial NO synthase, haemoglobin and endothelium-derived hyperpolarising factor are deeply involved in the increase of intravascular NO.

## 1. Introduction

Humanity is facing a great turning point in terms of its evolution. The novel coronavirus disease 2019 (COVID-19) pandemic caused by severe acute respiratory syndrome-coronavirus 2 (SARS-CoV-2) has become a global health emergency. According to the COVID-19 situation report published by the World Health Organization, on 30 May 2020, more than >5.8 million confirmed cases and 362,705 deaths across the world had been attributed to COVID-19 [1]. The explosive rise in the number of cases may overwhelm hospitals and collapse healthcare systems. The global lockdown of >3.5 billion people (almost half the global population) may disrupt the economy, businesses and employment in unprecedented ways. Many countries have also deployed digital surveillance tools to prevent the spread of COVID-19 without implementing government surveillance mechanisms, which may entail a drift towards a regime of authoritarian or totalitarian surveillance and control [2]. COVID-19 could lead to both the collapse of healthcare systems and societal destruction, including at the levels of the economy, politics, culture, households and the individual.

Nevertheless, paradoxically, COVID-19 has become an opportunity to discover new possibilities that could enable us to avoid total destruction. The lockdown does not necessarily mean loss. Indeed, we have witnessed rapid shifts to working from home, the acceleration of automation, the implementation of robots, the implementation of universal basic income and many other innovations. There has also been some early evidence that the lockdown policy has reduced global air pollution and global CO_2_ emissions [3,4]. COVID-19 has let humanity reach a new level of consciousness and led many to question why there is a world and how and why we live our lives. The fact that many people are facing their deaths entails that the world is collectively having a near-death experience [5] that can significantly increase self-esteem, mindfulness and the sense of the purpose of life [6,7]. Nevertheless, the direction of global society remains uncertain in the absence of great global leadership. Choices made by people and governments over the next few months will shape the world for years or even decades to come [2]. To produce social capitals in civil society structures, we need to establish balances between their closeness and openness, integration and linkage, human abilities and social capacities [8] without inclining towards division and isolation. We believe that self-reflection and the search for one’s true self followed by great collective leadership, information sharing, dialogue and cooperation at local and global levels are required to overcome the COVID-19 pandemic, as well as realising the true happiness of the members of all generations, which we believe is the goal of goals.

Among many social capacities, COVID-19 researchers have vividly demonstrated their good conscience and collective leadership for information sharing and cooperation at the global scale. A growing number of reports in the literature have discussed virology, clinical and molecular epidemiology, diagnosis, pathogenesis and potential therapeutics for the treatment and prevention of COVID-19 infection [9,10,11,12,13]. Similar to other RNA viruses, SARS-CoV-2 is characterised by significant genetic variability and a high recombination rate that enables its easy distribution among humans and animals worldwide [14]. Zhao et al. defined 17 viral subtypes along the SARS-CoV-2 genome sequences and showed how the regional distribution of subtypes can be used to track the progress of the pandemic [15], while van Dorp et al. identified 198 filtered recurrent mutations in the SARS-CoV-2 genome [16]. SARS-CoV-2 infects the host using the angiotensin converting enzyme 2 (ACE2) receptor, which is the sole receptor for entry [17,18]. ACE2 receptors are widely expressed in the heart (endothelium of coronary arteries, myocytes, fibroblasts and epicardial adipocytes), vessels (vascular endothelial and smooth cells), intestine (intestinal epithelial cells), lung (tracheal and bronchial epithelial cells, type 2 pneumocytes and macrophages), kidney (the luminal surface of tubular epithelial cells), testis and brain [19,20,21,22,23]. Accumulating evidence suggests that a subgroup of patients with severe COVID-19 acute respiratory distress syndrome (ARDS) with hypoxemia might have cytokine storm syndrome [24,25]. Indeed, 20–30% of COVID-19 patients showed abnormal blood clotting [26]. Blood clots can break apart and lodge in the lungs, resulting in pulmonary embolism, whereas clots from arteries can lodge in the brain, causing stroke. While the lungs are “ground zero”, clots can form in organ systems from the brain to blood vessels [25]. Varga et al. found evidence of direct SARS-CoV-2 infection of endothelial cells and diffuse endothelial inflammation [27]. Since SARS-CoV-2 targets blood vessels, patients with pre-existing damage to those vessels, such as from diabetes and high blood pressure, face a higher risk of serious disease. Although primary studies have reported similar respiratory diseases to SARS and MERS [28], vascular complications are rapidly emerging as a key threat of COVID-19.

There are currently no promising therapeutic strategies for either the treatment or prevention of COVID-19, despite the urgent need. While trials on SARS-CoV-2 genome-based specific vaccines (e.g., messenger ribonucleic acid (mRNA)-1273 by Moderna, Inc.; an adenovirus type-5 vector vaccine called Ad5-nCoV by CanSino Biologics, Inc.; a DNA-based vaccine called INO-4800 by Inovio Pharmaceuticals, Inc. and Beijing Advaccine Biotechnology Co.; an adenoviral vector-based vaccine called ChAdOx1 nCoV-19 or AZD1222 by AstraZeneca Plc., the University of Oxford and its spinout Vaccitech Ltd.; a lentiviral minigene vaccine called LV-SMENP-DC and lentiviral vector modified artificial antigen presenting cells (aAPCs) by Shenzhen Geno-Immune Medical Institute) [29] and therapeutic antibodies are currently being tested, these solutions are more long-term as they require thorough safety testing (e.g., the estimated study completion date of mRNA-1273 is 1 June 2021). Repurposing existing drugs previously designed for other virus infections and pathologies is also being examined as a rapid response measure to the emergent pandemic as the safety of most of these agents has already been tested. The agents can be divided into two categories depending on their target [14]. One acts directly on the coronavirus, either by inhibiting the crucial viral enzyme responsible for genome replication (RNA-dependent RNA polymerase inhibitors such as remdesivir, favipiravir, ribavirin, sofosbuvir, galidesivir and tenofovir and viral protease inhibitors such as lopinavir/ritonavir, azithromycin, ivermectin and nelfinavir) or by blocking viral entry to human cells (virus–cell membrane fusion inhibitors such as APN01 (rhACE2), chloroquine/hydroxychloroquine, teicoplanin and umifenovir). However, hydroxychloroquine and azithromycin combination could be lethal to COVID-19 patients [30]. The other is designed to modulate the human immune system, either by boosting the innate antiviral immune response (immunotherapies using natural killer cells and recombinant interferon) or by alleviating damage induced by dysregulated inflammatory responses (anti-inflammatory therapies using mesenchymal stem cells, intravenous immunoglobulin, anti-C5 monoclonal antibodies (e.g., eculizumab, siltuximab, TZLS-501, sarilumab and tocilizumab), anti-VEGF-A monoclonal antibody (bevacizumab), anti-TNF monoclonal antibody (adalimumab), SARS-CoV-2-specific neutralising antibodies, thalidomide, methylprednisolone and fingolimod). Passive immunotherapy, using recovered patients’ plasma, has been recommended for severe and critical cases of COVID-19, based on the presence of neutralising antibodies against SARS-CoV-2; however, concerns have been raised related to the potential risk for transfusion-transmitted infection and potential risk for severe disease due to antibody-dependent enhancement [31,32]. Various other treatments and prevention strategies are also currently under investigation, including oxygen therapies such as hyperbaric oxygen, nitric oxide (NO) and extracorporeal membrane oxygenation, convalescent plasma therapy, anticoagulant therapy, low-dose corticosteroid therapy, ACE inhibitors, angiotensin II type I receptor blockers, statins, antibiotics, polyclonal antibodies and other pharmacological agents. Natural compounds including traditional herbal medicines [33,34] and nutritional interventions such as vitamins, omega-3 fatty acids, selenium, zinc and iron [35] could also be considered for enhancing host immunity against COVID-19 infection. The application of big data analysis is also emerging to systematically identify the potential agents for drug repurposing against SARS-CoV-2 [36]. Both agent efficacy and agent safety, including in terms of adverse drug reactions and drug–drug interactions, await further clinical confirmation. Previous studies have proven the potential of several countermeasures, yet large-scale trials with a broader perspective remain necessary to respond to COVID-19.

The present study proposes an additional strategy for the treatment and prevention of COVID-19 by applying NO. In the vascular system, NO is produced by the endothelium, a single layer of cells that forms the inner lining of all blood vessels [37]. Endothelium-derived NO has several different functions, one of which is vascular smooth muscle relaxation, resulting in vasodilation and a consequent decrease in blood pressure and increase in local blood flow [37]; these discoveries were awarded the Nobel Prize in 1998 [38,39,40]. Previous studies treating SARS-CoV-infected patients with inhaled NO have shown pharmacological and antiviral effects of NO [41,42,43], suggesting that NO inhalation might be effective for COVID-19 patients [37,44]. Indeed, two clinical trials of inhaled NO are being conducted in COVID-19 patients [45,46]. Meanwhile, balneotherapy (hot spring therapy), sauna therapy and thermal therapy (hyperthermia) also reportedly increase endothelial NO synthase (eNOS), thus inducing vascular endothelial NO production [47,48,49,50,51,52]. However, hyperthermia may cause health issues such as heat stroke and cardiac morbidity. For example, there are 14,000 deaths in Japan per annum during bathing, which accounts for >15% of all sudden out-of-hospital deaths [53], while there are countries in which neither such therapies nor NO inhalation are accessible. One potential breakthrough in terms of overcoming these existing challenges may be nanomedicine or nanoscience, which have been advancing since the 1990s. Different classes of nanomaterials such as polymeric nanoparticles or nanocapsules, liposomes, metallic nanoparticles, metal oxide nanoparticles, silica-based nanoparticles, quantum dots, peptide or carbon nanotubes and up-conversion nanoparticles have been used for controlled NO delivery in medical applications [54]. However, this requires introducing NO-releasing nanomaterials into the human body, and the use of nanomaterials has become controversial due to health risks associated with their applications [55]. Meanwhile, a natural-mineral-based novel nanomaterial that is expected to have an effect similar to that of NO therapy without its intake into the body has been available since 2018. It was developed from Japanese hot springs and named integrated functional mineral crystal (IFMC) by Teikoku Pharmaceutical Co., Ltd., Japan [56]. If it were able to sufficiently increase intravascular NO, it might present a supportive treatment and prevention option for COVID-19 since it is inexpensive and easy to use (just putting it or spraying it in a solution on the body). Here, we demonstrate how the natural-mineral-based novel nanomaterial can induce an increase of intravascular NO, vasodilation and the consequent increase in blood flow.

## 2. Materials and Methods

### 2.1. Study Design and Participants

The experimental object of this research is the natural-mineral-based novel nanomaterial IFMC by Teikoku Pharmaceutical Co., Ltd., Osaka, Japan [56]. A solution of IFMC consists of haematite (Fe_2_O_3_), olivine (Mg_2_SiO_4_ and Fe_2_SiO_4_), rhodolite (MnCO_3_), zincite (ZnCO_3_) and additives such as deionized water, ethanol, methylparaben and sodium metabisulphite. The physical characteristics of IFMC were first investigated using a zeta-potential and particle size analyser, as well as scanning electron microscopy (SEM) coupled with an energy-dispersive X-ray Spectrometer (EDS). Then, the intravascular NO concentration was measured continuously using a NO sensor in the hepatic portal of rats to test whether IFMC could induce an increase of intravascular NO. Since the long-term continuous measurement of intravascular NO was impossible, complementary tests were conducted to determine whether IFMC could increase the surface temperature, blood flow rate, velocity and vessel diameter in the human body.

All experimental human and animal protocols were respectively reviewed and approved by the Medical Research Ethics Committee (Approval No. 413) and Animal Experimentation Committee (Approval No. 956) of Tokyo City University, Japan. All participants provided voluntary written informed consent prior to participation. We conducted a monitoring test prior to the present study. No problem was found in the monitoring results when spraying IFMC solution on the body or when wearing clothes impregnated with IFMC over several months. Since the naturally occurring substances that make up IFMC are hot-spring ingredients, which are completely different from chemical substances that may have mutagenic properties, we believe there would be no side effects for small animals or humans, even if they were taken directly.

### 2.2. Zeta Potential and Particle Size Measurement

The particle size distribution in the IFMC solution was measured using a zeta-potential and particle size analyser (ELS Z-2, Otsuka Electronics Co., Ltd., Osaka, Japan). Dynamic light scattering (photon correlation method) and the CONTIN method were used for the measurement. The measured temperature was 22.5 °C when the scattering angle was 165°. The result showed no scattering intensity. The possible reasons for this include: (1) there were no particles; (2) the particles were too small to measure (ELS Z-2 cannot measure particles smaller than 0.5 nm); (3) substances were present in the solution as ions and were therefore not present as minute particles. We think that mineral components exist as ions in IFMC solution since crystals were obtained when it dried up.

### 2.3. Field Emission Scanning Electron Microscopy and Energy-Dispersive X-Ray Spectroscopy

SEM images of IFMC were taken using an ultra-high-resolution cold-field emission scanning electron microscope FE-SEM (SU8230, Hitachi High-Tech Co., Tokyo, Japan), and EDS spectra were obtained using an energy-dispersive X-ray spectroscope (AZtecEnergy X-Max150, Oxford Instruments, Oxfordshire, U.K.). IFMC was dropped onto a microscope slide with a pipette and desiccated using a hot plate. The dried deposit was then secured to an FE-SEM carbon tape. The measurement conditions of FE-SEM were twofold: (1) magnification, ×100–5000; accelerating voltage, 1 kV; (2) magnification, ×100,000–200,000; accelerating Voltage, 10 kV. A silicon-drift detector was used to collect fluorescence spectra.

### 2.4. The In Vivo Measurement of Intravascular NO Concentration Using a NO Sensor in the Hepatic Portal Vein of Rats

A catheter-type NO sensor (INC-020, Inter Medical Co., Ltd., Aichi, Japan) was used with an integrated working electrode and a reference electrode (diameter, 0.5 mm; sensitivity, 500 pA μm^−1^). The electric current generated by an electrochemical reaction of NO was fed into a head amplifier (HAEC-A, B ×1/×10 or ×100/×1000, Inter Medical Co., Ltd., Aichi, Japan) and measured using an electrochemical amplifier (IMEC-601, Inter Medical Co., Ltd., Aichi, Japan) with the voltammetry method. IMEC-601/601A is currently the most improved measurement system for NO, oxygen, dopamine, glutamine acid and vitamin C both *in vivo* and *in vitro* [57,58,59,60,61].

Female Slc-Wistar rats (8–10 weeks) were obtained from Japan SLC, Inc. All rats were housed individually and maintained on an alternating 12 h light/dark cycle at 23 ± 1 °C. After a five day acclimatisation period, the rats were randomly divided into positive (use of IFMC solution; n = 2 rats for three samples) and negative control (placebo, i.e., with normal saline solution; n = 2 rats for six samples), with ad libitum access to drinking water. The small number of samples was due to the necessity of special surgical techniques; if this failed, the rats would quickly die due to heavy bleeding. Therefore, the present study limited the number of samples to secure the experiment from potential failures. All rats were anaesthetised using a cocktail of butorphanol tartrate (2.5 mL), midazolam (2.0 mL), medetomidine hydrochloride (1.875 mL) and physiological saline solution (3.625 mL) injected intraperitoneally at 2 mL kg^−1^ body weight and were maintained in their anaesthesia-induced unconscious state throughout the experiment. The NO sensor was inserted into the hepatic portal where blood vessels enter and leave the liver (portal vein, hepatic artery), bile ducts, lymph ducts and nerves all pass. This site was chosen because changes in intravascular NO concentration can be measured directly and stable measurement of arterial blood flow can also be made without damaging the artery itself. The chests of the rats in both groups were covered by Kimwipes^®^ with 5 mL IFMC solution or normal saline solution to test whether IFMC induces an increase of intravascular NO. Statistical significance was estimated based on the increase of intravascular NO concentration.

### 2.5. Measurements of Blood Flow Rate, Velocity and Vessel Diameter of the Human Brachial Artery Using an Ultrasonic Flow Meter

An ultrasonic Doppler blood flow meter (QFM-21, Hadeco Inc., Kanagawa, Japan) was used to measure the blood flow rate, velocity and vessel diameter of the human brachial artery non-invasively. The measurement conditions were as follows: ultrasonic frequency, 5.0 MHz (blood flow velocity measurement) and 7.5 MHz (blood vessel diameter measurement); ultrasonic output, ≤1500 mW cm^−2^ (peak time average output), ≤350 W cm^−2^ (peak pulse output), and ≤550 W cm^−2^ (maximum output); blood flow range, 0.15–377 mL s^−1^; blood flow rate, 5–120 cm s^−1^; blood vessel diameter, 2–20 mm; depth, 4–35 mm; blood flow rate measurement accuracy, ±10%. The research participants were four males aged 21–25 years. The measurements were conducted 22 times in total. The upper arm of each participant was covered by IFMC-added or IFMC-free textiles (100% polyester) to test whether IFMC could increase the blood flow rate, velocity and vessel diameter of the human body. Statistical significance was determined using a two-sided paired t-test.

### 2.6. Measurement of Human Hand Surface Temperature Using Thermography

The research participant was a 22-year-old female who felt coldness of the hands and feet on a regular basis. The measurement was started right after the solution of IFMC was sprayed on the surface of the participant’s hand. Changes in the hand surface temperature were measured using an auto-range thermo tracer (TH9100MR, Nippon Avionics Co., Ltd., Tokyo, Japan) at 1 min intervals during a total measurement period of 10 min. Statistical significance was determined based on pixel values using a two-sided paired t-test.

## 3. Results

### 3.1. SEM Image of IFMC

**Figure 1:**
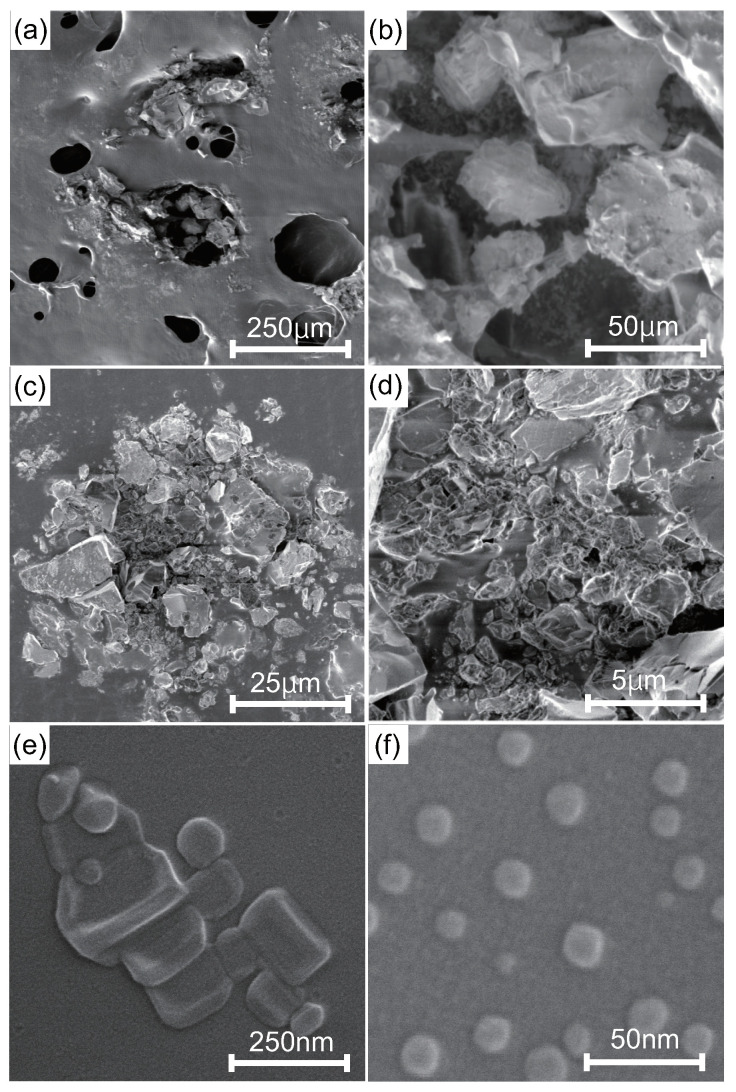
FE-SEM images of IFMC: (**a**) ×100 at 1 kV, (**b**) ×500 at 1 kV, (**c**) ×1000 at 1 kV, (**d**) ×5000 at 1 kV, (**e**) ×100,000 at 10 kV and (**f**) ×200,000 at 10 kV.

**Figure 2:**
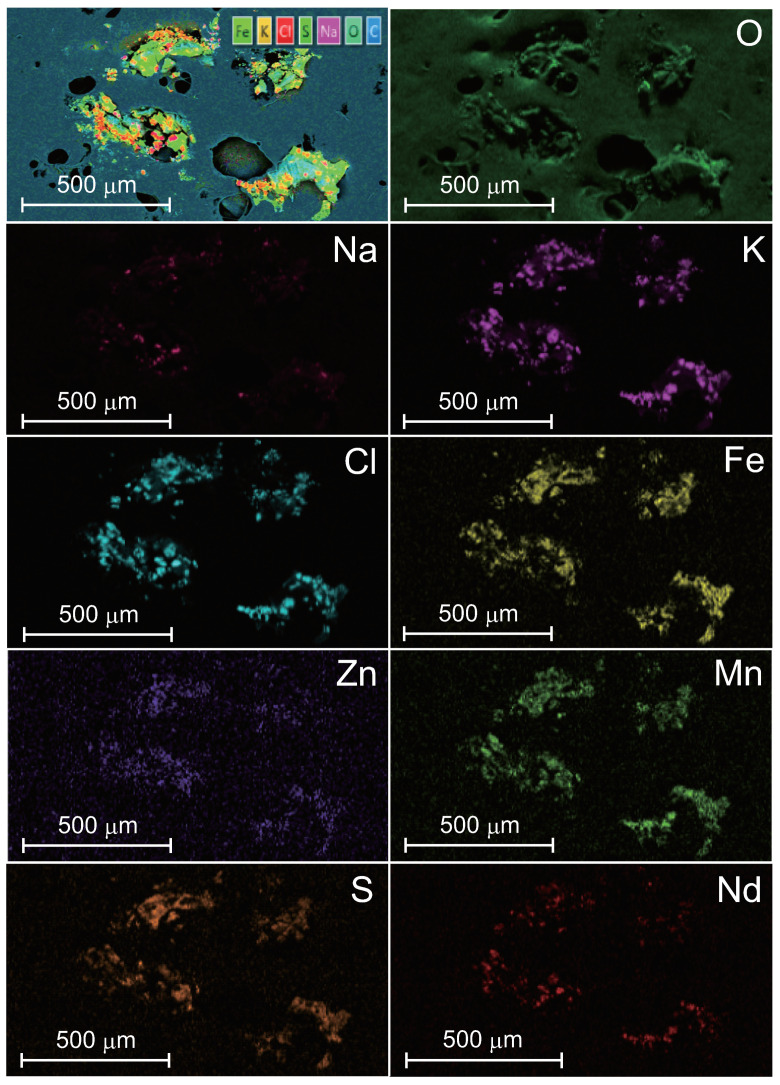
EDS element mapping corresponding to the SEM image of Fig. 1a. Elements include oxygen (O), sodium (Na), sulphur (S), potassium (K), chlorine (Cl), manganese (Mn), iron (Fe), zinc (Zn) and neodymium (Nd).

Figure 1 shows SEM images of IFMC. We observed different sizes and forms of crystals, including nano-sized crystals. Figure 2 shows element mapping images. The distribution of sulphur (S), manganese (Mn), iron (Fe), zinc (Zn) and neodymium (Nd) originating in mineral components extracted from natural minerals was confirmed. The concentration in descending order was as follows: O, Cl, K, Na, Fe, Zn, Mn, S and Nd.

### 3.2. Changes in Intravascular NO Concentration before and after IFMC and Placebo Applications

**Figure 3:**
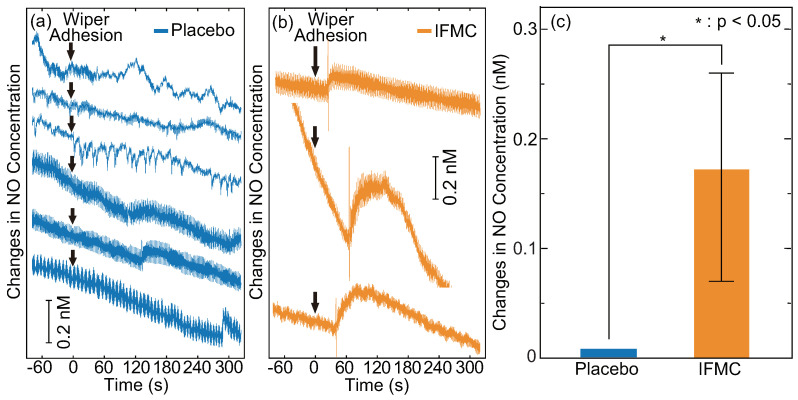
Changes in intravascular NO concentration before and after (**a**) placebo and (**b**) IFMC applications. The vertical axes are arbitrary scales that show the relative intravascular NO concentrations due to the different zero-offset values of each measurement. (**c**) shows the statistical significance of the increase of intravascular NO concentration, which was calculated as the difference between the minimum and maximum values after the wiper adhesion. The error bar indicates the standard deviation. The symbol * stands for P < 0.05.

Figure 3 shows changes in the intravascular NO concentration of rats before and after IFMC and placebo applications. The intravascular NO concentration started to increase 43.6 s on average after IFMC application with a range of 24.6–66.6 s. It increased by 0.07–0.26 nM with an average of 0.17 nM over 35.2 s on average (range, 17.4–47.4 s) and then gradually decreased. The peak value and duration of the increase were comparable to those of other reports, which used acetylcholine [62,63,64,65]. The lower peak value and shorter duration of the increase in this study were probably due to the small size of the rats. The minor spikes right before the increase were probably because of the relaxation of the vascular smooth muscle. Indeed, a similar spike was observed when the sensor was moved slightly. The result is, to our knowledge, the first evidence of inducing an increase of intravascular NO by bringing the natural-mineral-based nanomaterial into contact with or close to a living body without pharmacological intervention or physical intervention (e.g., acupuncture, massage therapy or balneotherapy).

### 3.3. Differences in Blood Flow Rate, Velocity and Vessel Diameter in the Brachial Artery between IFMC and Placebo Applications

**Figure 4:**
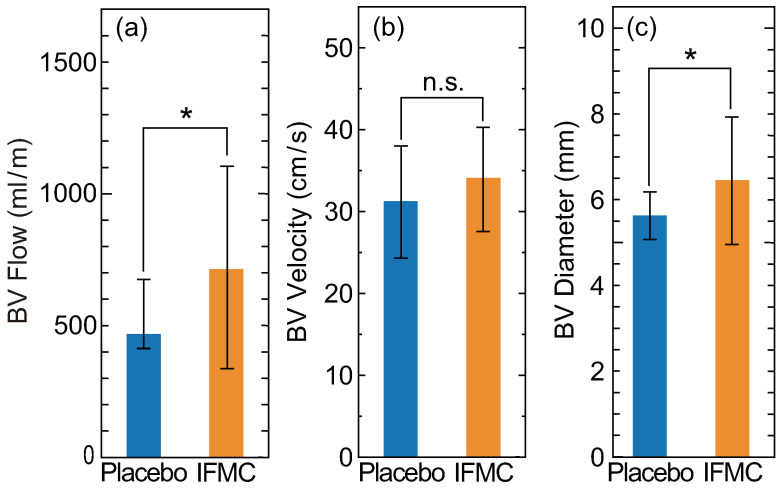
Differences in (**a**) blood flow rate, (**b**) velocity and (**c**) vessel diameter in the brachial artery between IFMC and placebo applications. The upper arm of each participant was covered by IFMC-added or IFMC-free textiles. The error bars indicate the standard deviation. The symbols * and n.s. stand for P < 0.05 and P > 0.05, respectively.

Figure 4 shows changes in the blood flow rate, velocity and vessel diameter in the brachial artery. The ranges of the systolic and diastolic peaks of each parameter were comparable to the values of another report [66]. Compared with IFMC and placebo applications, significant increases were observed in blood flow rate and vessel diameter, but there was no significant difference in blood flow velocity. Statistical significance values of blood flow rate, velocity and vessel diameter between placebo and IFMC applications were 0.0188, 0.1911 and 0.0387, respectively. The significant increases in blood flow rate and vessel diameter were due to vasodilation derived from the increase in intravascular NO. The minor change in blood flow velocity was due to the improvement of vascular elasticity followed by relaxation of vascular smooth muscle. The result (i.e., the increase of the blood flow rate due to vasodilation without blood flow velocity change) indicates improvement of the neuroimmunoendocrine system [67,68,69].

### 3.4. Changes in Hand Surface Temperature after IFMC Application

**Figure 5:**
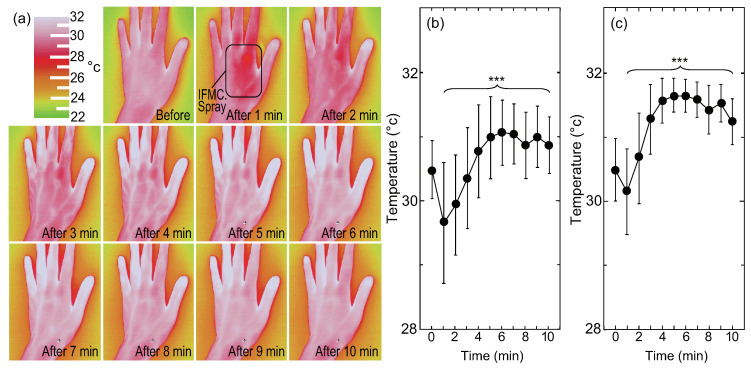
Changes in (**a**) hand (back) surface temperature before and after IFMC application observed by thermography. The right figures show the changes in the surface temperature of the (**b**) entire hand and (**c**) fingers before and 10 min after IFMC application. The error bars indicate the standard deviation. The symbol *** stands for P < 0.001.

Figure 5 shows changes in the surface temperature of a hand (back) observed by thermography. Before spraying the IFMC solution, the mean surface temperature of the entire hand (back) was 30.5 °C, and that of the fingers was 30.5 °C, indicating that the peripheral circulation was poor. After 1 min, the surface temperature decreased due to vaporisation. However, 2 min later, some of the relatively thick blood vessels in the hand began to swell, and after 3 min, almost all of the thick blood vessels on the back of the hand stood out. Meanwhile, after 3 min, the temperature of the peripheral blood vessels in the fingertips increased. The surface temperature of the area close to the blood vessels rose. The temperature of the entire hand had increased at 5 min. The clear behavioural transition from thick to thin blood vessels was obviously due to vasodilation. The mean surface temperature of the entire hand (back) was 30.9 °C and that of the fingers 31.2 °C at 10 min. The differences in the surface temperature of the entire hand and fingers before and 10 min after IFMC application were significant. The increase in the fingers (+0.9 °C) was larger than that in the entire hand (+0.4 °C). The difference is evidence of vasodilatation and the increase of blood flow rate in the peripheral blood vessels.

## 4. Discussion

The present study confirmed that the natural-mineral-based novel nanomaterial IFMC, with a size of tens of nanometres (Figure 1), could induce an increase of intravascular NO (Figure 3), vasodilation (vessel diameter) and blood flow rate in a living body (Figure 4), as well as an increase of the surface temperature of a hand including fingers (Figure 5). Despite the lower peak value and shorter duration of the increase in intravascular NO concentration (Figure 3) due to the small size of the rats, which were comparable to those of other reports [62,63,64,65], the increase of human hand surface temperature lasted at least 10 min (Figure 5), suggesting that the effect of IFMC was significant. Further, the fact that the increase of the mean surface temperature of the fingers (+0.9 °C) was larger than that of the entire hand (+0.4 °C) at 10 min after IFMC application (Figure 5) suggested that the vasodilatation and increase of blood flow rate in the peripheral blood vessels were caused by NO as an endothelium-derived relaxing factor (EDRF). This is corroborated by the increase in the diameter of blood vessels in the brachial artery and the increase in blood flow (Figure 4). The results are, to our knowledge, the first evidence of inducing an increase of intravascular NO and vasodilation by bringing the natural-mineral-based nanomaterial into contact with or close to a living body, without pharmacological intervention or physical intervention (e.g., acupuncture, massage therapy or balneotherapy).

The precise mechanism of the induction of intravascular NO remains uncertain; however, there are two possibilities. The primary possibility is NO synthesis. It is well known that the activity of eNOS followed by NO production in the vascular wall is regulated by receptor-mediated signalling, such as vascular endothelial growth factor receptor 2 (VEGFR2) and acetylcholine (ACh), as well as protein partners, including heat shock protein 90 (HSP90), calmodulin (CaM) and caveolin-1 (Cav-1) [70]. The receptor-mediated biological response includes a time lag between actions of chemical mediators (messenger substances) and receptor reaction. In contrast, IFMC caused its effect within 1 min (Figure 2 and Figure 5). The quick response indicates the possibility that IFMC principally involves another mechanism, not receptor-mediated action.

Therefore, we assume haemoglobin (Hb) is deeply involved in the phenomenon. It is known that hypoxic vasodilation does not require the synthesis of EDRF/NO [71]. Hb and NO independently fulfil diverse and complex physiological roles, while together, they subtly modulate microvascular perfusion in response to second-by-second changes in local metabolic demand, contributing to hypoxic vasodilation [72]. Among the candidate molecular mechanisms, only S-nitrosohemoglobin (SNO-Hb) directly fulfils the physiological requirements [72]. Thus, NO is transported by red blood cells to microvascular sites of action in protected form as an S-nitrosothiol on the highly conserved Hb β-93 Cys residue, invariant in birds and mammals [72]. Meanwhile, NO as a vascular EDRF is a gaseous signal substance and considered to be freely diffused between vascular endothelial cells and vascular smooth muscle cells. However, NO does not freely diffuse between the endothelial cells and smooth muscle cells of blood vessels [70,73]. The oxidation state of Hb is harnessed to control how much NO reaches the smooth muscle [74]. Hb has the structure of haem iron with four iron porphyrin units combined with a globin protein. Hb has the ability to combine with oxygen molecules (O). Therefore, Hb not only plays a role of transporting oxygen (O_2_), but also is deeply involved in the control of NO transport. It is well known that the transport of O_2_ and NO in blood is facilitated by the spin transition of iron atoms in Hb. The ferrous ions in haem iron are five-coordinated high-spin complexes; however, they become six-coordinated low-spin complexes when oxygen molecules are added. Ferrous ions move to the centre of the porphyrin ring by changing the spin state of *d*-electrons from high to low spin. This movement at the molecular level changes the structure of Hb to enhance the affinity or adsorbability of the other three haem-iron complexes to oxygen molecules. In other words, the electronic structure of *d*-electrons controls the uptake and release of oxygen molecules. Therefore, we may form the following hypothesis: the affinity of Hb for NO might be reduced by bringing IFMC into contact with or close to a living body.

In light of this, magnetic evaluation using a magnetic probe such as a positive muon may clarify the mechanism of action by IFMC on Hb. Indeed, the mixture of Hb as an organic compound and IFMC as an inorganic nanomaterial, which contains transition metal compounds exhibiting antiferromagnetism and weak ferromagnetism, may trigger several different types of interactions. One of the potential methods of clarifying the mechanism is to estimate the magnitude of the internal magnetic field that the muon experiences when IFMC is irradiated with muon beams. Specifically, we would be able to examine the relation between the spin properties of IFMC through muon experiments using a high-energy accelerator and the spin transition changes of iron atoms involved in the changes in the affinity or adsorbability to the O/NO molecule of haem iron in Hb. Through the experiments, it would be possible to examine IFMC’s remote action or action over a distance between atoms and atoms or between atoms and molecules. In the fields of semiconductor engineering and spintronics, there are previous studies claiming that simple antiferromagnetic insulators may transmit spin information over a distance of several tens of micrometres [75]. The proposed examination might be the world’s first experiment to uncover the spin transmission over a much longer distance.

Meanwhile, the potential benefits of IFMC are probably not limited to the induction of intravascular NO. It is known that there are three different endothelium-derived vasorelaxation factors involved in maintaining vascular homoeostasis: prostacyclin (PGI2), NO [38,39,40,76] and endothelium-derived hyperpolarising factor (EDHF) [77]. They are all derived from vascular endothelium, whereas they differ in their production processes: NO is produced from arginine by eNOS; EDHF is synthesised by a variety of stimulants, such as agonists, shear stress and acetylcholine in a Ca^2+^/calmodulin-dependent manner [78]; and prostacyclin is produced by cyclo-oxygenase and prostacyclin synthase enzymes [79]. It has been shown that NO and EDHF fulfil complementary roles. NO mediates vascular relaxation of relatively large conduit arteries (e.g., aorta and epicardial coronary arteries), whereas EDHF plays an important role in modulating vascular tone in small, resistance arteries (e.g., small mesenteric arteries and coronary microvessels) [80]. The vascular tone can be regulated by an EDHF-like mechanism in the absence of NO [81]. The clear behavioural transition from thick to thin blood vessels (Figure 4) suggests that IFMC influences not only the induction of intravascular NO, but also the production of EDHF. In addition, the effect of IFMC on neuroimmunoendocrine system improvement, indicated by the increase of blood flow rate due to vasodilation without blood flow velocity change (Figure 5), is also worthy of further examination. Thus, the precise mechanism and other potential benefits of IFMC are possible future research topics.

However, the limited findings of the present study provide insight into the treatment and prevention of COVID-19. The increase of intravascular NO (Figure 3) can be expected to have both pharmacological and antiviral effects. The pharmacological actions of NO are twofold: vascular and nonvascular smooth muscle relaxation and regulation of airway function and the pathophysiology of inflammatory airway disease [82]. NO has also been shown to have antiviral actions, such as an inhibitory effect on the synthesis of viral protein and RNA, including the replication cycle of SARS-CoV [41,42,43]. In general, the action mechanism of inhaled NO has been thought to be pulmonary vasodilation and the consequent improved oxygenation in the blood of the lungs, which kills the virus, as it does not do well in a high-oxygen environment. Ignarro further added that NO also interacts directly with the virus to kill it and/or inhibit its replication, and NO inhalation is more effective as an antiviral agent than simply as a vasodilator in pulmonary circulation [37]. The proposed natural-mineral-based novel nanomaterial might be added as an supportive treatment and prevention option for COVID-19, since it is inexpensive and easy to use (just putting it or spraying it in a solution on the body). Given its advantages, it has the potential to overcome the limitations of inhaled NO therapy, such as the cost and availability of devices and skilled medical staff. The health risks of the existing NO-releasing medical nanomaterials might no longer be a matter of concern, since IFMC does not require intake into the human body. It is probable that the increase of intravascular NO concentration (Figure 3) is not abnormal; however, quantitative examination of the increase and its persistence in the human body is necessary. Nevertheless, there is still an urgent need for promising therapy strategies for both the treatment and prevention of COVID-19.

## 5. Conclusions

To summarise, our inter- and trans-disciplinary approach revealed that the natural-mineral-based novel nanomaterial IFMC can induce an increase of intravascular NO, vasodilation and blood flow rate, as well as an increase of hand surface temperature in a living body. Therefore, it might become a supportive treatment and prevention option for COVID-19. The precise mechanisms remain a matter for further investigation; however, we may assume that eNOS, Hb and EDHF are deeply involved in the increase of intravascular NO. The remaining research agendas are to (1) examine if IFMC can induce an increase of intravascular NO and improve long-term blood circulation in humans, (2) clarify how intravascular NO is induced by bringing IFMC into contact with or close to a living body, (3) examine IFMC’s remote action or action over a distance between atoms and atoms or between atoms and molecules, (4) explore other potential benefits of IFMC, (5) explore the possibility of other natural-mineral-based nanomaterials, (6) clinically examine whether IFMC is effective in the treatment and prevention of COVID-19 and (7) explore urgently and collectively a series of promising therapy strategies for both the treatment and prevention of COVID-19 based on larger scale trials from a broader perspective.

## Abbreviations

The following abbreviations are used in this manuscript:

ACE2	angiotensin converting enzyme 2
ACh	acetylcholine
aAPCs	artificial antigen presenting cells
ARDS	acute respiratory distress syndrome
CaM	calmodulin
Cav-1	caveolin-1
Cl	chlorine
COVID-19	novel coronavirus disease 2019
EDHF	endothelium-derived hyperpolarising factor
EDRF	endothelium-derived relaxing factor
EDS	energy-dispersive X-ray spectrometer
eNOS	endothelial nitric oxide synthase
Fe	iron
FE-SEM	field emission scanning electron microscopy
Hb	haemoglobin
HSP90	heat shock protein 90
IFMC	integrated functional mineral crystal
K	potassium
Mn	manganese
mRNA	messenger ribonucleic acid
Na	sodium
Nd	neodymium
NO	nitric oxide
O	oxygen
PGI2	prostacyclin
S	sulphur
SARS-CoV-2	severe acute respiratory syndrome-coronavirus 2
SEM	scanning electron microscopy
SNO-Hb	S-nitrosohemoglobin
VEGFR2	vascular endothelial growth factor receptor 2
Zn	zinc
